# Game-based training improves the surgeon’s situational awareness in the operation room: a randomized controlled trial

**DOI:** 10.1007/s00464-017-5456-6

**Published:** 2017-03-09

**Authors:** Maurits Graafland, Willem A. Bemelman, Marlies P. Schijven

**Affiliations:** Department of Surgery, Academic Medical Centre, PO Box 22660, 1100 DD Amsterdam, The Netherlands

**Keywords:** Videogame, Medical education, Smartphone, Minimally invasive surgery, eHealth, Cholecystectomy

## Abstract

**Background:**

Equipment-related malfunctions directly relate to one-fourth of the adverse events in the surgical theater. A serious game trains residents to recognize and respond to equipment problems in minimally invasive surgery (MIS). These include disturbed vision, gas transport, electrocautery, and pathophysiological disturbances. This randomized controlled trial explores whether game-based training improves surgical residents’ response to equipment-related problems during surgery.

**Methods:**

Thirty-one surgical residents with no previous experience in MIS took part in a standardized basic laparoscopy training course. Fifteen residents were randomly assigned to the game-enhanced curriculum (intervention) and sixteen were assigned to the regular curriculum (control). Participants performed a MIS task in a live anesthetized pig model, during which three standardized equipment malfunction scenarios occurred. Observers recorded the problems recognized and solved, time, and participants’ technical performance.

**Results:**

Twenty-four participants completed the post-test (*n* = 12 per group). The intervention group solved more problems than the control group (59 vs. 33%, *p* = 0.029). The intervention group also recognized a larger proportion of problems, although this parameter was non-significant (67 vs. 42%, *p* = 0.14). Random effects modeling showed a significant improved game performance per participant over time.

**Conclusions:**

Surgical residents, who play for only 1 h on a custom-made serious game, respond significantly better to equipment-related problems during surgery than residents trained by a standard training curriculum. These results imply that entertaining serious games can indeed be considered for use in official training for surgeons and other medical specialists.

Minimally invasive surgery (MIS) has been widely adopted in various surgical procedures, reducing overall patient morbidity whilst improving cosmetic results. However, the surgeon’s increased workload in a technology-dependent environment [1] increases the chance for errors to occur. Errors relating to the equipment occur frequently in the laparoscopic suite and pose a significant threat to patient safety [2–5]. A recent systematic review shows that equipment malfunctions are to be held responsible for nearly a quarter of the adverse events in the OR [2].

Standardized MIS training courses aim to develop knowledge and psychomotor skills and are part of surgical training in many countries [e.g., fundamentals of laparoscopic surgery [6] (FLS)]. Basic laparoscopic training courses focus on laparoscopic principles and dexterity training. However, they do not educate surgical trainees to deal with the laparoscopic environment or with equipment-related errors. Even experienced professionals seem to be insufficiently equipped to solve laparoscopic equipment-related problems, when they encounter them during MIS [7]. Recent studies show that long-term knowledge preservation regarding MIS equipment after basic laparoscopy courses is poor [8]. Additional training focused on long-term knowledge retention and dealing with non-routine events during MIS is therefore much needed.

A serious game (Dr. Game, Surgeon Trouble^®^) was developed to train surgical personnel in recognizing and solving equipment-related problems in MIS. Playing this specifically developed serious game is likely to improve trainees’ problem recognition and problem-solving skills in the OR. The serious game’s construct validity was established in a previous study [7]. Serious games are digital applications that are both fun to play and supply the player skills, knowledge, or attitudes useful in reality [9]. Both virtual reality simulators [10] and serious games [11] have proven to be effective modalities to improve surgeons’ laparoscopic dexterity and suturing skills. Serious games enhance voluntary play among trainees compared to virtual reality simulators, which make them interesting training solutions for busy professionals [12].

This study examines the influence of this custom-made serious game on surgical trainees’ problem recognition and problem-solving capabilities during equipment malfunctions in the laparoscopic OR. We hypothesize that trainees who follow a game-enhanced curriculum would recognize and solve more equipment-related problems than trainees who followed the regular basic laparoscopic training curriculum.

## Methods

### Study design

This randomized, single-blinded controlled trial was conducted at a tertiary academic center in the Netherlands. The institutional ethics committee has reviewed the study protocol and concluded that full review was unnecessary because it is not a clinical trial. The institutional animal studies review board approved the study.

### Participants

Participants were residents in their first or second year of general surgical training participating in the standard basic laparoscopic training course (BLTC). They were required not to have any experience in MIS as a primary surgeon. After giving consent, participants were enrolled into either the control group (regular BLTC curriculum) or the intervention group (game-enhanced BLTC curriculum). Randomization was conducted using a sealed opaque envelope with equal probability of group allocation. Participants could not be blinded due to the nature of the intervention.

### Setting: basic laparoscopic training course

The BLTC is an obligatory part of the surgical residency training curriculum in the Netherlands [13] and is based on FLS principles [6]. The purpose of the course is to familiarize novice surgical trainees with laparoscopic principles, equipment, and basic dexterity. The 2-day curriculum consists of lectures on the principles of laparoscopic instrumentation, laparoscopic tower, pneumoperitoneum, electrocautery and vessel sealing, ergonomics, cholecystectomy, appendectomy and hernia repair, and technical skills training on a laparoscopic box trainer (peg transfer, cord placement, rubber band placement and cutting, and cholecystectomy on a cadaver liver), after which trainees complete a hands-on interactive training session on a live anesthetized pig model (trocar positioning, cholecystectomy, appendectomy).

### Intervention

**Fig. 1:**
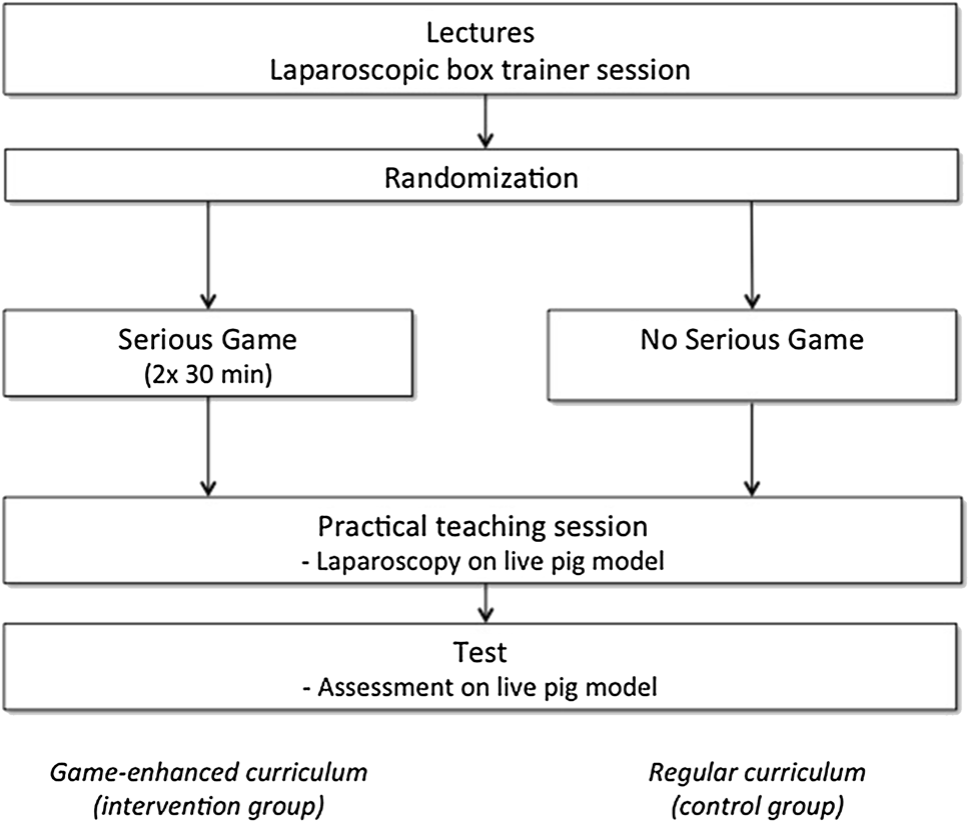
Curriculum followed by both study groups in the basic laparoscopy training course

The game-enhanced curriculum consisted of the regular BLTC, enhanced by two separate 30-min sessions of serious gaming, containing an estimated 10 play sessions (Fig. 1). The participants received an individual login and standardized instruction tutorial before commencing the game. The control group followed the regular BLTC curriculum. They had the opportunity to explore the laparoscopic equipment during the intervention groups’ gaming sessions.

### Serious game

**Fig. 2:**
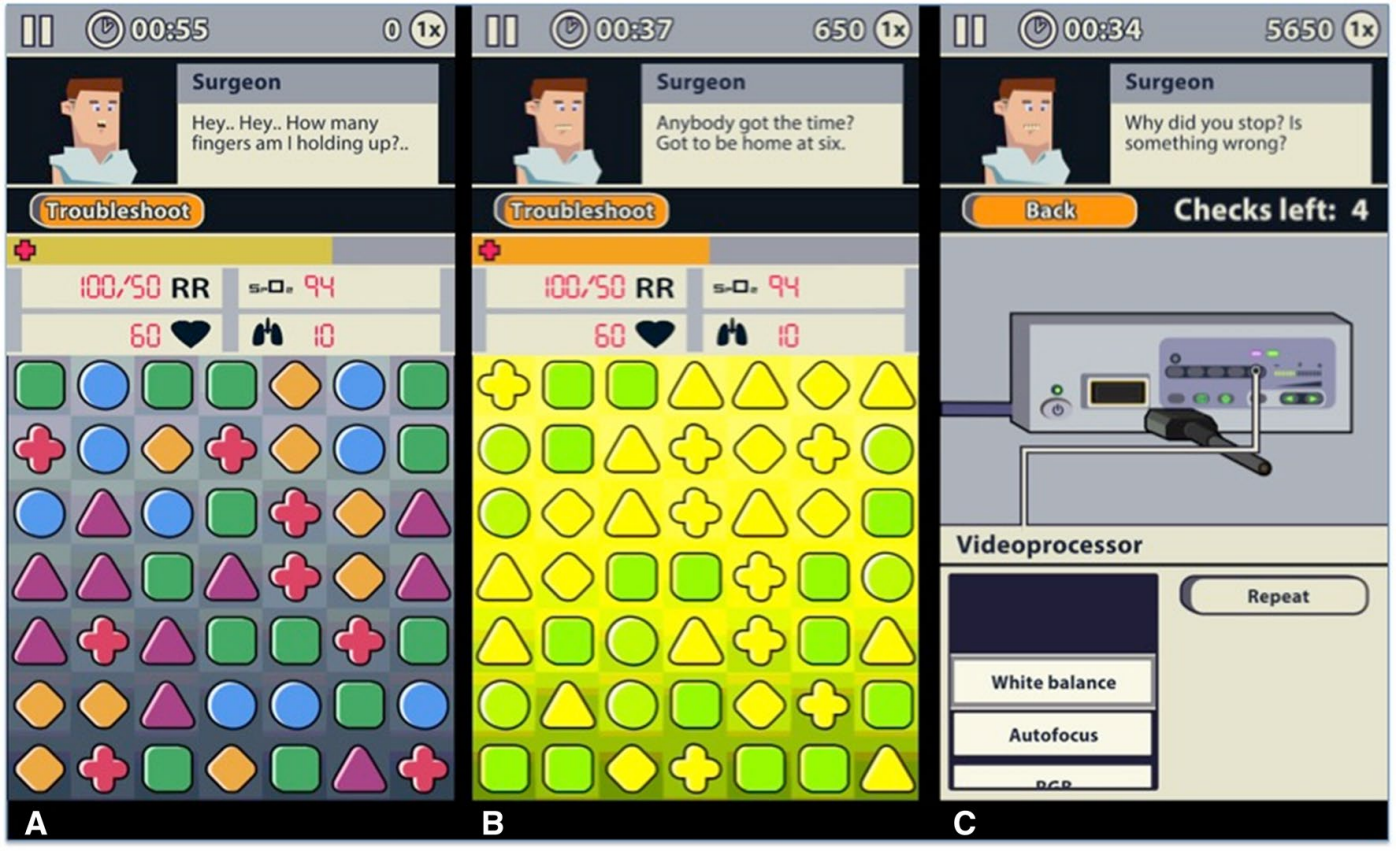
The serious game (*screenshots*). **A** Main screen, with mini-game (*below*), the patient’s vital signs, and a supervising surgeon (*above*). **B** During the mini-game, the player deals with problem scenarios that resemble real-life problems in MIS, for example the *blurred screen*. **C** After the player recognizes the problem scenario, he or she can solve it by selecting the correct action on a simulation of the MIS equipment

Dr. Game, Surgeon Trouble^®^ (WeirdBeard co., Amsterdam, The Netherlands) was designed to train surgical trainees in recognizing and responding to equipment problems of the laparoscopic tower [7] (Fig. 2). The game consists of a entertaining mini-game designed to attract the player’s attention. The main objective in the game is to create rows of three similar blocks—which is fun and challenging (Fig. 2A). The task, although not requiring any professional expertise, demands the player’s full attention. Meanwhile, the laparoscopic tower is virtually embedded in the gameplay, and in order to progress, one must be able to solve laparoscopic equipment-related problem scenarios. Changes in the environment (screen, sounds, values) signify equipment-related problems and malfunctions. Signals partly occur outside the player’s direct focus of attention, similar to the OR environment. The player scores extra points by timely recognizing the problem, after which he or she enters a troubleshooting mode in the game (Fig. 2B).

The player can solve the problem by selecting the correct equipment and actions, after which he or she can play again and ‘score’ again. The player receives direct feedback. This creates a continuing cycle of challenges, actions, and feedback. It can be expected that the player’s problem-solving ability in the laparoscopic operation room will improve by playing this specific serious game. Secondly, dealing with these situations in a game environment may also familiarize novice surgeons with the principles of situation awareness. The participants’ game performance is measured and stored in a database (the amount of scenarios recognized and solved, time required, and the amount of correct/incorrect actions required to solve the problem).

The game’s educational content includes problem scenarios of screen and lighting (19), gas transport and pneumoperitoneum (5), electrocautery (2), and pathophysiological disturbances related to MIS (2). Content has been previously validated by equipment specialists. The game screen relates to the camera and lighting, handling blocks to the electrosurgical unit, and the appearance of the visual field to the pneumoperitoneum. Per 3-min game session, the player encounters approximately six problem scenarios. The ‘troubleshooting mode’ (Fig. 2C) depicts a laparoscopic tower (Olympus Exera II CLV 180 light source, Olympus UHI-3 insufflator, Olympus Exera II CV-180 video processor, EndoEYE HD Video Laparoscope, and SurgMaster UES-40 electrosurgical unit (all: Olympus co., Tokyo, Japan)). The simulated MIS unit in the game corresponds to the unit used in the BLTC.

### Outcome assessment

All participants performed two standardized tasks on a live anesthetized pig model as the primary surgeon, consisting of (1) searching the small bowel for a Meckel’s diverticulum and (2) performing a biopsy of the parietal peritoneum. During their procedure, they were assisted by two OR nurses and a camera navigator. Three standardized equipment problem scenarios occurred: (1) failure of the insufflation and pneumoperitoneum, (2) failure of the electrocautery unit, and (3) saturation change on the anesthesia monitor (Table 1).

**Table 1:** Participants encountered three standardized problem scenarios during the final assessment

Problem scenario	Cause	Symptoms	Timing	Correct steps
(1) Insufflator malfunction	Gas tank closed upon start	- Alarm insufflator (auditory)	From start	- Check insufflator
				- Check gas tank
		- Loss of pneumoperitoneum		- Check gas tubes
		- Insufflator gas bar empty		- Check trocar position and valves
(2) Electro-surgery malfunction	Patient grounding plate not fit	- Alarm (auditory)	From start electrocoagulation task (±3 min)	- Check display electrosurgical unit
				- Check cables
		- Electrocoagulation failure		- Check patient grounding plate
(3) Pulse saturation change	Pulse oximeter malfunction	- Auditory pulse signal fails to appear	Simultaneous with scenario #2	- Check anesthesia monitor
		- Flat line anesthesia monitor		- Check pulse oximeter

Primary outcome measures were the proportion of problems recognized and solved per participant; secondary outcome measure was the time required to do so. These measures were calculated independently from each other. An independent assessor, blinded to group allocation, registered these parameters. Problem recognition was defined as the participant verbally or otherwise indicating that a problem had occurred <2 min after the onset of the “symptoms,” and problem solving was defined as solving it <2 min after problem recognition.

An experienced surgeon blinded to group allocation assessed the participants’ technical skills through an Objective Structured Assessment of Technical Skills (OSATS) form. This contains 7 items (tissue handling, movement, instrument handling, instrument knowledge, use of assistance, procedural progress, and procedural knowledge) scored on a 5-point Likert scale [14]. The participants received a standardized instruction before the test, during which they were told that they would be judged on their technical performance only (OSATS). They were instructed to use and coach the OR personnel present as they would normally do and talk aloud in case of trouble.

The participants’ learning curves in the game-enhanced curriculum group were calculated to assess if their performance during gaming sessions improved and, thus, if learning did occur (% of problems solved per individual game session).

### Sample size

Prior to the trial, a pilot study was performed in which eight surgical residents with no MIS experience as primary surgeon were assessed using the set-up described above. Using an alpha of 0.05, a power of 0.80, a population standard deviation of 0.186, and an estimated effect size of 50%, the required size for each group was 12. The dropout rate was estimated at 20%.

### Statistical analysis

Descriptive statistics were calculated for all variables. All data were not normally distributed and thus Mann–Whitney U tests were applied to calculate the differences in the primary and secondary outcome measures. Subgroup analyses were performed assessing the performance of both groups on the individual problem scenarios. Differences were calculated using Pearson Chi-square tests. To estimate the learning curves during the game-enhanced curriculum group’s individual game sessions, a random effects model was calculated. Analyses were performed using the IBM Statistical Package for Social Sciences version 20 (IBM corp., Armonk, NY, USA) and R version 2.15 (R Foundation for Statistical Computing, Vienna, Austria).

## Results

### Participant characteristics

**Fig. 3:**
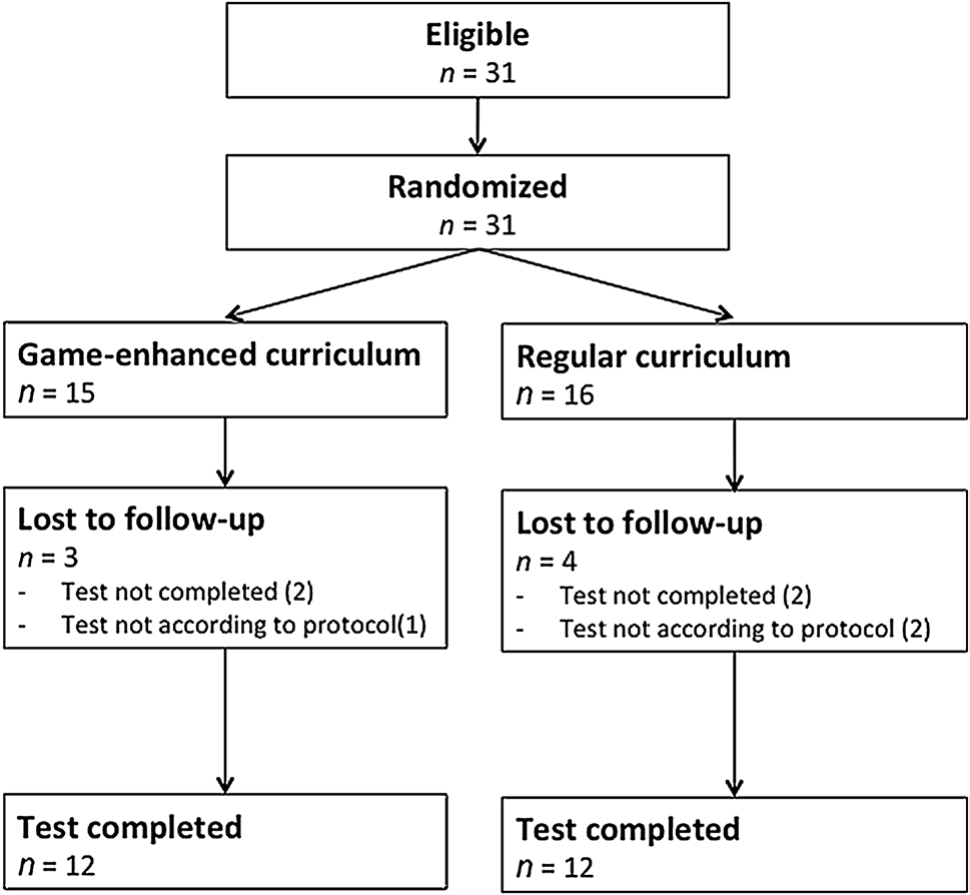
Flowchart of the participants through the study protocol

Thirty-one surgical residents were randomized between May 2013 and April 2015. In total, 24 completed the curriculum and the assessment according to protocol (12 per group, Fig. 3). Four participants did not complete the test because of an incident occurring at the test site, requiring it to be closed down. Three participants were removed from the analysis due to protocol violations. Disturbances during the test caused heterogeneity of surgical circumstances deviating beyond normal variability.

None of the participants had experience as primary surgeon in MIS. The game-enhanced curriculum group completed an average of 11.8 game sessions, relating to about 60 min of gameplay. There were no differences between age, gender, technical score (OSATS), postgraduate year, or experience in conventional surgery. The regular curriculum group contained slightly more residents in their 2-year preparatory training for a specialty other than general surgery (e.g., orthopedic, plastic, cardiothoracic surgery, or urology). An overview of the demographic characteristics is shown in Table 2.

**Table 2:** Demographic characteristics

Demographic characteristics		Game-enhanced curriculum	Regular curriculum
Group size	*n*	12	12
Age	Mean, SD	29.4 (±1.7)	28.8 (±1.2)
Gender	M	58.3%	58.3%
	F	41.7%	41.7%
Residency curriculum	General surgery	5/12	3/12
	Preparatory training	7/12	9/12
Technical skills (OSATS)	Median score (1–5) IQR	2.4 (2.2–3.2)	2.8 (2.2–3.2)
Postgraduate year	1st	1	2
	2nd	10	10
	3rd	1	0
Experience in MIS (as primary surgeon)		0/12	0/12
Experience in non-MIS procedures (as primary surgeon) *n* = 21	None	3/12	3/12
	1–20 procedures	5/12	4/12
	21–50 procedures	4/12	2/12
Play sessions completed (on serious game)	Mean, SD	11.8 (±1.7)	–

Preparatory training: 2-year general surgery training incorporated in residency curricula, orthopedic, cardiothoracic, plastic surgery, and Urology

*IQR* Interquartile range, *MIS* minimally invasive surgery, *OSATS* objective structured assessment of technical skills, *SD* standard deviation

### Primary and secondary outcome parameters

**Fig. 4:**
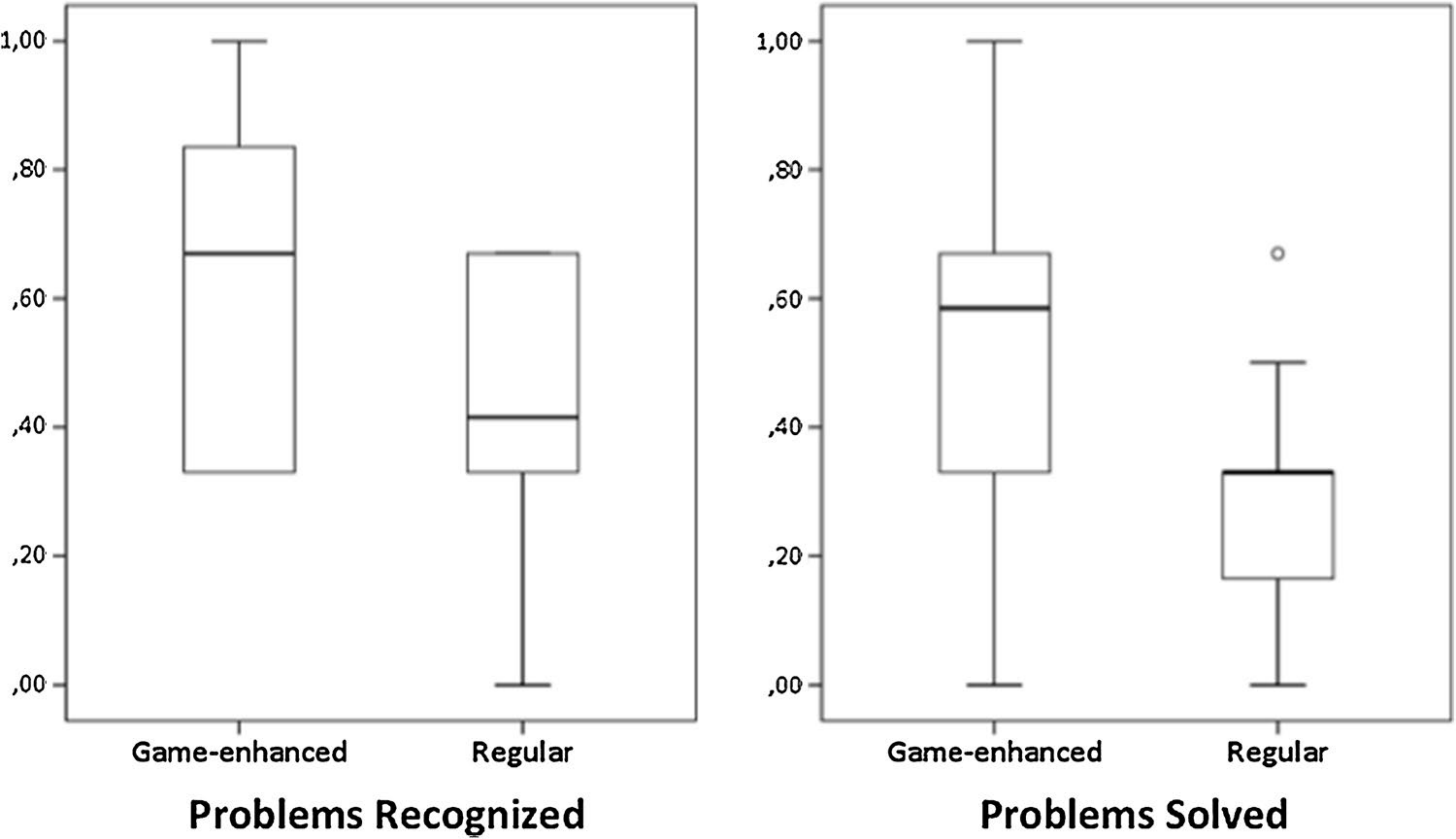
Problems recognized and solved in game-enhanced and regular curriculum groups. *Boxes* depict median and interquartile range, and the error bars represent the 90% range

The participants in the game-enhanced curriculum group solved a median of 59% (interquartile range (IQR) 33–67%) of the problems presented to them, compared to 33% (8–33%) in the regular curriculum group (Fig. 4, *p* = 0.03). Participants in the game-enhanced curriculum group recognized a median of 67% of the problems (IQR 33–92%), compared to 42% (33–67%) in the regular curriculum group (Fig. 4, *p* = 0.14).

The game-enhanced curriculum group recognized problems in a median of 66 s (IQR 52–85 s) vs. 80 s (68–86 s) in the regular curriculum group, *p* = 0.24. The game-enhanced curriculum group solved a problem in a median of 75 s (IQR 46–88 s) vs. 85 s (75–101 s) in the regular curriculum group, *p* = 0.14.

### Subgroup analysis

A subgroup analysis of problem types recognized and solved by intervention and control groups is shown in Table 3. The difference in the total number of problems solved between the intervention and control groups was statistically significant (20/36 vs. 11/35, *p* = 0.04). The most obvious difference between the intervention and control groups was observed in the participant’s ability to recognize (8/12 vs. 4/11, *p* = 0.14) and deal with insufflator malfunctions (8/12 vs. 3/11, *p* = 0.06).

**Table 3:** Subgroup analysis: problems recognized and solved as specified by each group

	Problem type	Game-enhanced curriculum (intervention)	Regular curriculum (control)	p*
Recognized	Insufflator malfunction	8/12 (67%)	4/11 (36%)	0.14
	Electrocautery malfunction	12/12 (100%)	12/12 (100%)	N/A
	Saturation change	3/12 (25%)	0/12 (0%)	0.21
	Total	23/36 (64%)	16/35 (46%)	0.12
Solved	Insufflator malfunction	8/12 (67%)	3/11 (27%)	0.06
	Electrocautery malfunction	10/12 (83%)	8/12 (67%)	0.35
	Saturation change	2/12 (17%)	0/12 (0%)	0.14
	Total	20/36 (56%)	11/35 (31%)	0.04

*Chi-square test

### Learning curve

**Fig. 5:**
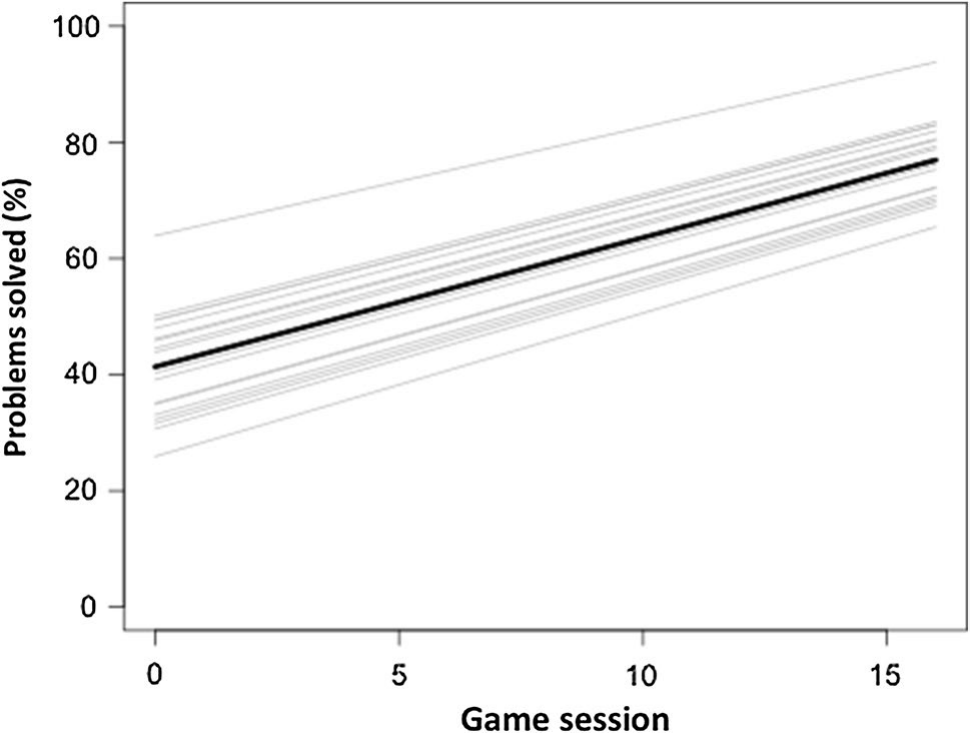
Estimated learning curve of naïve players per game session. *Gray lines* depict the estimated learning curves per participant (*n* = 12) and *black line* depicts the estimated average

The participants in the game-enhanced curriculum group each completed a mean of 11.8 game sessions (SD 1.7), in which they played a mean of 63.9 problem scenarios (SD 13.0). In the first four game sessions, they solved a mean of 48.1% of the problems (SD 14.5), in the second four sessions 54.5% (SD 9.9), and in the third four sessions 69.3% of the problems (SD 14.5). Their learning curve during the game sessions was estimated using a linear regression model with random intercepts (Fig. 5). This shows a 2.3% improvement in the proportion of solved cases per three-min session (*p* < 0.001).

## Discussion

This randomized controlled trial effectively demonstrates that 1 h of practice on a custom-made serious game results in an improved problem-solving performance concerning equipment-related problems in the MIS theater. The importance of training laparoscopic equipment failure scenarios is emphasized by the relatively poor performance of the control group, showing that the current laparoscopy training courses are insufficient to train pupil’s ability to recognize and respond to non-routine events in the MIS environment. Participants in the game group were not only able to solve more equipment-related problems, but also more likely to solve equipment failure problems that did not occur in their area of focus (e.g., insufflation of the pneumoperitoneum and vital parameters), indicating an improved situational awareness. This has considerable implications for both the surgical training curriculum and patient safety in the OR.

Serious gaming is an innovative training method that is currently being explored in medical pre- and postgraduate training [15–17]. A well-designed serious game appeals to the intrinsic motivation of the trainee to play, while educational content is fitted in a subtle, ‘stealthy’ fashion [18]. Through repeated, voluntary interaction with the content, games lead to experiential learning [19]. Although the effectiveness of serious games to enhance ‘technical’ surgical skills has been shown in previous studies [20–22], this is the first study in which a serious game is systematically assessed for its capacity in training the ability to anticipate non-routine adverse events in the surgical theater.

Although both primary outcome parameters (problems recognized and solved) show a reasonable effect size, only the latter is statistically significant. The range in participants’ ability to recognize problems was larger than the range in the ability to solve problems, which accounts for the difference in statistical significance.

Logically, a higher number of participants would have led to statistical significance in this parameter too. A trend is also seen in the time required by the participants to recognize and solve problems in favor of the intervention group. This parameter is of lesser importance to novice learners, whereas acting haphazardly is more dangerous in the OR than acting slow and consciously.

Other studies have proven the effectiveness of serious games for laparoscopic psychomotor skills training (‘technical’ skills). Jalink et al. compared the performance of surgeons and non-surgeons on a specifically developed Wii™-based serious game and a laparoscopic box trainer, finding a significant, high correlation [20]. Badurdeen et al. found similar correlations between performance on Wii™-based entertainment games and laparoscopic box trainer scores [21]. Youngblood et al. compared the training results of medical students in trauma management between a serious game and patient simulator, finding a significant, comparable improvement in skills in terms of a behavioral performance evaluation scale [23]. To our knowledge, this study is the first randomized study to prove the effect of a serious game in terms of performance improvement in the surgical environment (i.e., *predictive* validity).

A second strength is that the serious game intervention was applied within the regular curriculum. Participants in the control group participated in the customary BLTC, which includes lectures on laparoscopic instrumentation and the laparoscopic tower. These lectures include handling specific equipment-related problem scenarios. This substantially increases the generalizability of the study results, whereas the systematic game-enhanced curriculum and the regular curriculum are compared and not merely a “trained” and a “non-trained” group.

A limitation of this study is the relatively high dropout rate (22.5%). Although 31 participants were initially recruited, only 24 completed the post-test according to the protocol, equaling the minimum required number in the power analysis. A higher inclusion number was deliberately obtained because of suspected high dropout rates based on literature [24]. All participants failed due to logistical reasons and none refused to partake in the test. The test protocol was complex, relying heavily on the performance of the study personnel in staging the test setting and equipment failure scenarios. This led to protocol violations in four occasions. Ultimately, group sizes and baseline characteristics (technical performance, previous surgical experience, and demographic characteristics) did not differ significantly. Selection bias due to dropout therefore seems limited. Future research on situational awareness in the OR should limit the complexity of the study protocol and reliance on trained personnel.

A second limitation is that retention of learning in time was not measured. Due to the use of live animal models and participants in clinical employment in multiple teaching hospitals, repeated measurements would have posed great logistical challenges. From a practical point of view, one may state that, whereas this study proves that gameplay improves performance, continued gameplay is likely to maintain this level of skill.

The serious game used in this study is unique in the sense that the gameplay resembles a popular arcade-type animated game, in which important surgical content was embedded. Whereas most currently available medical serious games apply realistic graphical simulations to mimic reality [15], this animated approach has deliberately been chosen to preserve interest of the player, which is especially novel to the field. It has been shown that high graphical fidelity to the medical construct (e.g., near-perfect graphical depiction of the operation room) is not necessary to teach important medical content, as long as the game’s *functional* fidelity (e.g., resemblance of important ‘cues’ in the action or procedure) remains adequate [25]. This implies that future surgical training does not necessarily have to take place in realistic e-learning or simulation modules, but could be delivered through entertaining and attractively animated videogames. Simple and compelling games are known to be fun, reinforcing, and even addictive [26]. This aims to captivate the user and improve the interaction time.

The place of serious gaming in the surgical residency curriculum is somewhat ambiguous. Its main advantage is the ability to invoke ‘voluntary play’ by using motivational triggers such as competition and attractive gameplay [12]. This distinguishes serious games from less challenging simulators, which are frequently left untouched by trainees, unless they are obligated [27]. As the optimal effect of serious games is reached through the trainee’s intrinsic motivation (‘voluntary play’), ‘obligatory play’ of serious games in the surgical curricula thus seems to be a contradiction in terms. However, because non-routine events training carries clinical importance in terms of patient safety, the achievement of a minimally required level of expertise for trainees seems inevitable.

## Conclusions and recommendations

This randomized controlled trial shows that surgical trainees that follow a game-enhanced curriculum have a significantly higher ability to solve equipment-related problems in the MIS theater than surgical trainees that follow the regular curriculum. Equipment failure is known to lead to procedural delays and represents a potential threat to patient safety. Future research should determine the value of ‘voluntary play’ of serious games compared to an obligated minimally required level of performance and relate these findings to the long-term retention of performance.
